# Effect of the Teaching Nursing Home on Nursing Home Staff Satisfaction

**DOI:** 10.1093/geroni/igaf122.3754

**Published:** 2025-12-31

**Authors:** Howard Degenholtz, Nancy Zionts, Anny Treat, Kenna Campbell, Chelsea Dickson

**Affiliations:** University of Pittsburgh, Pittsburgh, Pennsylvania, United States; Jewish Healthcare Foundation, Pittsburgh, Pennsylvania, United States; University of Pittsburgh, School of Public Health, Pittsburgh, Pennsylvania, United States; University of Pittsburgh, School of Public Health, Pittsburgh, Pennsylvania, United States; Jewish Healthcare Foundation, Pittsburgh, Pennsylvania, United States

## Abstract

The Teaching Nursing Home Collaborative (TNHC) aims to enhance the long-term care workforce and improve care quality in nursing homes through clinical education, and quality improvement organized around the Institute for Healthcare Improvement’s Age-Friendly Health System “4Ms” framework: What Matters Most, Mobility, Mentation, and Medication. Nursing homes also participate in an online learning community. Staff satisfaction data were collected using the Centers for Medicare and Medicaid Services (CMS) Nursing Home Employee Satisfaction Survey from two nursing homes between 2022 and 2023, and three in 2024. A total of 726 surveys were available for analysis. In addition, two nursing homes that are part of the TNHC shared staff satisfaction survey data collected by commercial vendors. A subset of five items that also appear on the CMS Survey were analyzed for 2021 – 2024; the effective sample size for these five items was 1,317. The results were mixed. Overall job satisfaction decreased significantly at one nursing home (p=.0000), but did not change significantly at the other two facilities. For all five facilities, there was an increase in satisfaction related to team dynamics (i.e., cross-departmental p=.000 and supervisory engagement p=.0300). However, ratings declined significantly for items related to recommending the facility as a workplace, having adequate resources, and opportunities for professional growth (p < .0001). As the Age-Friendly Nursing Home program is promulgated nationally monitoring staff experience and satisfaction remains critical to sustaining workforce engagement and care quality.

